# Starfish Gonadotropic Hormone: From Gamete-Shedding Substance to Relaxin-Like Gonad-Stimulating Peptide

**DOI:** 10.3389/fendo.2019.00182

**Published:** 2019-03-25

**Authors:** Masatoshi Mita

**Affiliations:** Center for Advanced Biomedical Sciences, Research Institute for Science and Engineering, Waseda University, Tokyo, Japan

**Keywords:** gonadotropic hormone, gamete-shedding substance, gonad-stimulating substance, relaxin-like gonad-stimulating peptide, 1-Methyladenine, starfish

## Abstract

The first report of a gonadotropic substance in an invertebrate hot-water extract of radial nerve cords from starfish *Asterias forbesi* that induced the shedding of gametes when injected into the coelomic cavity in a ripe individual occurred in 1959. The active substance was named gamete-shedding substance (GSS) or radial nerve factor. GSS is the primary mediator of oocyte maturation and ovulation in starfish. However, the effect of GSS is indirect. Resumption of meiosis in immature oocytes and release from the ovary are induced by a second mediator, maturation-inducing hormone, identified as 1-methyladenine (1-MeAde) in starfish. The role of GSS is to induce 1-MeAde production by ovarian follicle cells. Thus, GSS was redesignated as gonad-stimulating substance (also GSS). Although GSS has been characterized biochemically as a peptide hormone, identification of the chemical structure had to wait until 2009. Fifty years after the initial finding, GSS was purified from the radial nerve cords of starfish *Patiria pectinifera (P. pectinifera)*. The purified hormone was a heterodimer composed of A- and B-chains, with disulfide cross-linkages. Based on its cysteine motif, GSS is classified as a member of the insulin/insulin-like growth factor (IGF)/relaxin superfamily. More specifically, phylogenetic sequence analysis revealed that *P. pectinifera* GSS is a member of the relaxin-type peptide family. Therefore, GSS in starfish has been redesignated as relaxin-like gonad-stimulating peptide (RGP). Subsequently, orthologs of *P. pectinifera* RGP have been identified in other starfish species, including *Asterias amurensis (A. amurensis)*, and *Aphelasterias japonica (A. japonica)*.

## Introduction

Regulation of reproduction differs among animals. This variety can appear more as a gathering of exceptions, and it is hard to explain reproductive regulation by only a single mechanism. In fact, the meiotic stage of oocytes for the timing of fertilization differs widely among animals. Sea urchin eggs have long been used as a principal material for the study of developmental biology, because both eggs and spermatozoa are obtained easily by injecting isotonic potassium chloride into the body cavity. In other words, sea urchin oocytes have already accomplished meiotic maturation within the ovaries during the breeding season long before spawning occurs. On the other hand, in starfish belonging to the same phylum as sea urchins, Echinodermata, meiosis is arrested at the prophase of the first meiotic stage in fully grown oocytes just before spawning. These immature oocytes within the ovary still possess a large nucleus (germinal vesicle [GV]) and fail to undergo fertilization even though oocytes isolated mechanically from ovaries are inseminated. In order to undergo normal fertilization, the immature oocytes must resume meiotic division just before spawning under the influence of an active substance. Thus, starfish are suitable animals for the study of regulatory mechanism of oocyte maturation and ovulation. It is also important that 1-methyladenine (1-MeAde) has been purified from starfish *A. amurensis* (1), and 1-MeAde on its own is capable of inducing oocyte maturation and ovulation. Therefore, 1-MeAde in starfish is the first maturation-inducing hormone (MIH) identified in the animal kingdom. Numerous studies about the hormonal role of 1-MeAde on oocyte maturation in starfish have been published to date (2–9). On the other hand, the chemical structure of the gonadotropic hormone of starfish remained unknown for 50 years, even though its activity had been found 10 years before the discovery of 1-MeAde. In 2009, starfish gonadotropic hormone was the first identified among invertebrates (10). In this review, the outcome of research on starfish gonadotropic hormone from its discovery to its identification is described.

## Gamete-Shedding Substance

Gonadotropic hormones play important regulatory roles in reproduction in both vertebrates and invertebrates. In vertebrates, two kinds of gonadotropins are secreted from pituitary glands: follicle-stimulating hormone (FSH) and luteinizing hormone (LH). These two glycoproteins are structurally and functionally conserved across various species. Glycoprotein hormones that include FSH, LH, thyroid stimulating-hormone (TSH) and thyrostimulin (TS) are widely distributed not only in vertebrates but also in invertebrates (11). However, it is unclear whether pituitary glycoprotein hormones are involved in reproduction in invertebrates.

In 1959, Chaet and McConnaughy (12) first reported that injecting a hot-water extract of the radial nerve cords of starfish *Asterias forbesi* induced the release of eggs and spermatozoa from ripe females and males, respectively. This finding opened the door to the study of reproductive endocrinology in asteroids. An active substance present in the radial nerve cords has been found in all starfish species so far tested (about thirty) (3). The extract prepared from male nerve cords can induce the release of gametes in both sexes (13). Not only whole animals (13–15) but also isolated gonads (16–18) respond to the radial nerve factor, so the active substance is considered to act directly on the gonads. Thus, the substance was first designated as gamete-shedding substance (GSS) (18).

**Figure 1:**
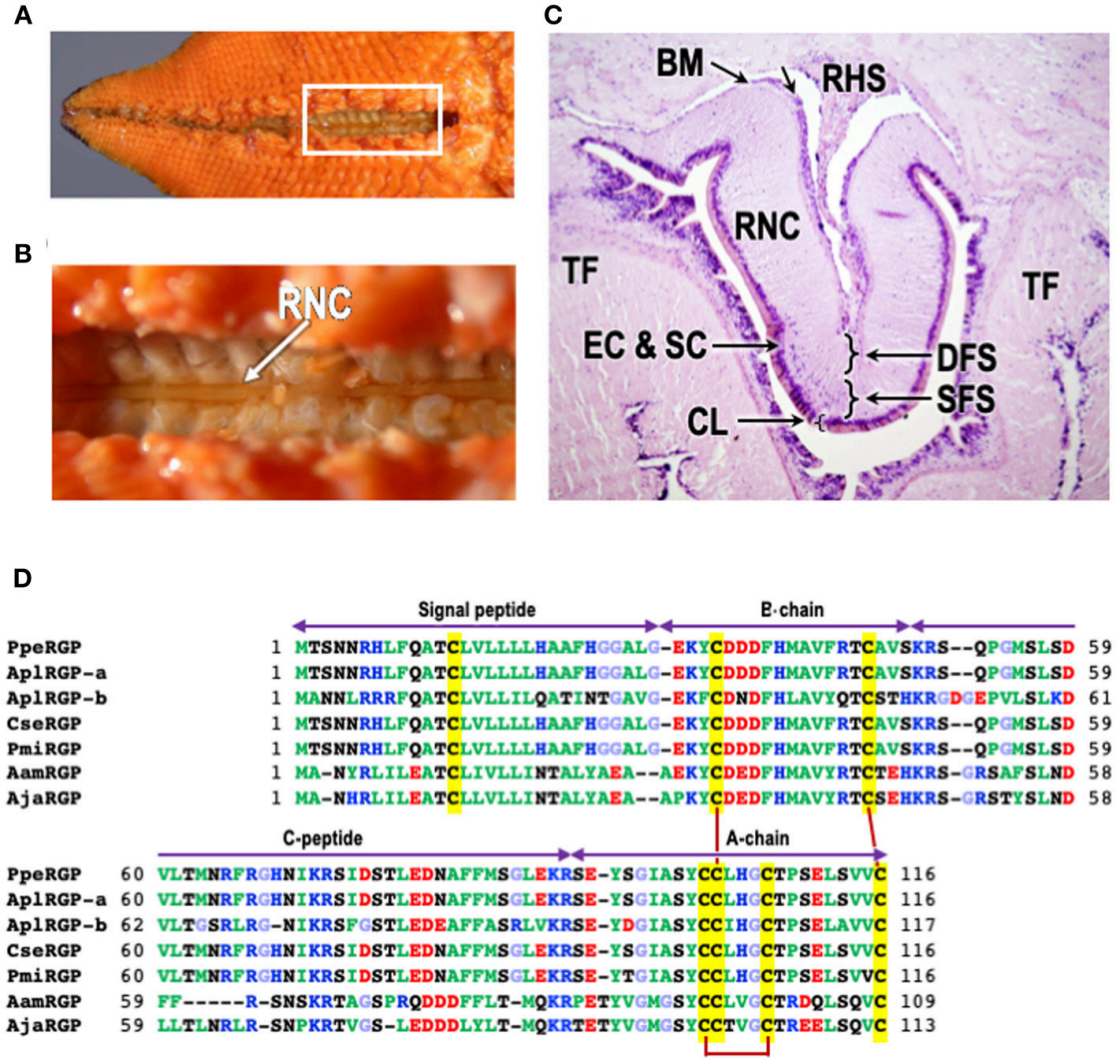
Structure of radial nerve cords of starfish *P. pectinifera* and chemical structures of relaxin-like gonad-stimulating peptides (RGP) in various species of starfish. **(A)** Aboral surface of *P. pectinifera*. **(B)** Magnified image of the area in the square in **(A)**. Arrow indicates radial nerve cords. **(C)** Cross-section of the tissue around radial nerve cord of *P. pectinifera*. The specimen was stained with hematoxylin-eosin. **(D)** Alignment of RGP pre-prohormone sequences of *P. pectinifera, Acanthaster planci (A. planci), Certonardoa semiregularis (C. semiregularis), P. miniata, Asterisa amurensis (A. amurensis)*, and *A. japonica*. The signal peptide, C-peptide, B- and A-chains are indicated. To illustrate the conserved features, the amino acid types are color coded according to their nature with basic residues in blue (Arg, Lys, and His), acidic residues in red (Glu and Asp), hydrophobic residues in green (Ala, Val, Ile, Phe, Trp, Tyr, Pro, and Met), hydrophilic in black (Ser, Thr, Asn, and Gln) and glycine in light blue. The cysteine residues are highlighted in yellow, and the disulfide bonds are shown by solid dark red lines. The abbreviations and the sequence sources [DDBJ accession numbers]: AamRGP, *A. amurensis* RGP (LC040882); AjaRGP, *A. japonica* RGP (LC104980); AplRGP-a, *A. planci* RGP-a (LC033566); AplRGP-b, *A. planci* RGP-b (19); BM, basement membrane; CL, cuticle layer; CseRGP, *C. semiregularis* RGP (LC066682); CT, connective tissue; DFC, deep fiber system; EC, epithelical cells; SC, sensory cells; PmiRGP, *P. miniata* RGP (LC057656); PpeRGP, *P*. (*Asterina*) *pectinifera* RGP (AB496611); RHS, radial heamal sinus; RNC, radial nerve cord; SFS, superficial fiber system; and TF, tube foot.

The content of GSS in the radial nerve cords (Figures 1A,B) has been shown to be equal in both sexes, when assayed with isolated ovarian fragments *in vitro* (20). GSS is also present in the radial nerve cords throughout the year and its quantity is mostly the same irrespective of the breeding season (17, 18). In individuals, GSS mostly exists in the radial nerve cords and circumoral-nerve rings (20). However, GSS is also abundantly found in various other parts of the body, such as the epidermis, tube-feet and cardiac stomach, although its activity is much less than in the radial nerve cords (20). It is important that GSS is detected in the coelomic fluid only when individuals are undergoing natural spawning (20). Thus, this indicates that GSS is a hormone.

Microsurgical procedures show that GSS is located mostly in the supporting cells located under the cuticle layer of the surface of the radial nerve cords (Figure 1C) (17, 21). Histological studies also indicate that GSS is contained in neurosecretory-like granules in the same area of the radial nerve cords (22, 23). Further electron microscopic observations of the radial nerve cords and on GSS-containing granules isolated by differential centrifugation and sucrose density gradient ultracentrifugation from a homogenate of radial nerve cords show that GSS is present in granules contained in the supporting cells (21). Similar granules are also found in the subepithelial plexus of tube-feet, body wall, and cardiac stomach where GSS activity is detected (20, 23). Although the pyloric caecum contains an extensive nerve plexus, neither GSS granules nor GSS activity are detected. According to the histological study by Unger (24), GSS seems to be transported from the supporting cells along the supporting fibers in radial nerve cords to the radial and transverse haemal sinus (Figure 1C), and then to the water vascular system, thus reaching the coelomic cavity where the gonads are suspended.

## Gonad-Stimulating Substance

Although GSS is the primary mediator of oocyte maturation and ovulation in starfish, the effect of GSS is indirect. The action of GSS is production of a second mediator, 1-MeAde, as an MIH in starfish (1–5). When GSS reaches the ovary, it enters the ovary and acts on follicle cells around oocytes to produce 1-MeAde (25, 26). 1-MeAde is found from the incubation mixture of GSS and ovarian fragments of *A. amurensis* (1), and 1-MeAde induces spawning when injected into the coelomic cavity of ripe starfish (27). Isolated ovarian fragments also undergo spawning in seawater containing 1-MeAde. Thus, the GSS (gamete shedding substance) has been redesignated as gonad-stimulating substance (still termed GSS) (28).

The shedding of spermatozoa begins after a shorter latent period than that of eggs after 1-MeAde treatment. The intervals preceding the discharge of gametes after the injection of 1-MeAde are almost equal to those found after injection of GSS. This indicates that 1-MeAde production in ovarian follicle cells begins immediately after GSS application (28–30).

Although GSS is thermostable, insoluble in organic solvents, and dialyzable, its activity is lost by treatment with proteolytic enzymes (14, 17, 31), indicating that GSS is a peptide hormone. Chaet (32) first tried to purify GSS from radial nerve cords of *Patiria miniata (P. miniata)*. The result suggested that GSS consisted of at least 42 amino acid moieties, implying a minimum molecular weight of around 4,800. In contrast, the GSS purified from the radial nerve cords of *A. amurensis* showed that GSS was a single peptide consisting of some 22 amino acids, with a molecular weight of about 2,100 (33). Since GSS is composed of two peptides combined by disulfide bonds (10), it might be possible that the disulfide bond of GSS is reduced during the purification process to become a single peptide. Despite active efforts, they could not determine the amino acid sequences of GSS, because the detection threshold of analytical instruments was insufficient at that time. It was also difficult to undertake physiological assays using chemically synthetic peptides.

## Relaxin-Like Gonad-Stimulating Peptide

It took 50 years since the initial finding by Chaet and McConnaughy (12) before GSS was finally purified from the radial nerve cords of starfish *Patiria* (synonym, *Asterina*) *pectinifera* and its chemical structure identified (10). For the purification of the GSS, 126.3 g wet weight of total radial nerve cords were collected from 5,500 animals (Figures 1A,B). The purification procedures are briefly described. After homogenization and extraction, the nerve extracts were applied to a four-step high-performance liquid chromatography (HPLC) procedure using a reverse-phase column. GSS activity was biologically assayed by ovarian fragments of *P. pectinifera*. After the 4th HPLC, GSS was finally purified and the amino acid sequence analyze using a protein sequencer and electronspray ionization-tandem quadruple/orthogonal-acceleration TOF MS/MS equipped with a nano-HPLC system. The purified hormone was a heterodimeric peptide with a molecular weight of 4,740 Da, comprising an A-chain of 24 amino acids (aa) and a B-chain of 19 aa, with disulfide cross-linkages; two interchain between the A- and B-chains, and an intrachain within the A-chain (10). The molecular weight of GSS in *P. pectinifera* was close to that of *P. miniata* estimated by Chaet (32). It is important that the A-chain contains a cysteine motif [CCxxxCxxxxxxxxC], which is a signature sequence of the insulin/insulin-like growth factor (IGF)/relaxin superfamily. Based on its cysteine motif, starfish GSS has been classified as a member of the insulin/IGF/relaxin superfamily. Furthermore, a phylogenetic tree of the insulin/IGF/relaxin superfamily strongly suggests that GSS belongs to the relaxin-like peptide family (10). The name of GSS had not adequately expressed characteristics of a peptide hormone. Thus, GSS has been designated once again as a relaxin-like gonad-stimulating peptide (RGP) (34).

Synthetic RGP induces oocyte maturation and ovulation in ovarian fragments of *P. pectinifera* within 30 min of incubation. A median effective concentration is approximately 1–2 nM (10). In contrast, neither oocyte maturation nor ovulation occurs when ovarian fragments are incubated with the A-chain alone, the B-chain alone, or a mixture of A- and B-chains. Additionally, spawning behavior and subsequent spawning can be seen after injection with synthetic RGP into the males and females of *P. pectinifera* with fully grown testes and ovaries, respectively (10).

The *RGP* gene in *P. pectinifera* (*PpeRGP*) consists of 3,896 base pairs (bp) comprising two exons and one intron. The lengths of exons 1 and 2 are 208 and 2,277 bp, respectively, with an intron of 1,411 bp between them (DDBJ: LC027939) (34). The transcript consists of 2,485 bases (b) in length (DDBJ: LC027938), although a size of the open reading frame (ORF) is 351 b (DDBJ: AB496611) (10). This indicates that only 14% of the *PpeRGP* mRNA is translated into the peptide. Also, PpeRGP is a very highly conserved peptide, because genetic variation and polymorphism have not been found in the *PpeRGP* gene among ten local populations from Japanese waters (35). The ORF of PpeRGP encodes a peptide of 116 aa, including a signal peptide of 29 aa at the N-terminus (10). The signal peptide is followed by the B-chain, and the A-chain is located at the C-terminus. There is an intermediate sequence (C-peptide) of 44 aa between the B- and A-chains, which have typical proteolytic cleavage sites, Lys and Arg, at the ends (Figure 1D). Thus, after the formation of three disulfide cross-linkages between the A- and B-chains and within the A-chain, mature RGP is produced by elimination of the signal and C-peptides.

The chemical structures of RGP in *A. planci* (AplRGP-a) (DDBJ: LC033566) (36), *A. planci* (AplRGP-b) (19), *C. semiregularis* (CseRGP) (DDBJ: LC066682) (35), and *P. miniata* (PmiRGP) (DDBJ: LC057656) (35) are almost the same as that of *P. pectinifera* (Figure 1D). Because *A*. *planci, C. semiregularis, P. miniata*, and *P. pectinifera* belong to the order Valvatida in the class Asteroidea, the chemical structure of PpeRGP is considered to be well-conserved among starfish of the order Valvatida beyond species (37).

In contrast, the chemical structures of RGP identified from *A. amurensis* (AamRGP) and *A. japonica* (AjaRGP) of the order Forcipulatida are quite different from that of PpeRGP (38, 39), although the cysteine motifs of AamRGP and AjaRGP coincide exactly with that of PpeRGP (Figure 1D). This suggests that AamRGP and AjaRGP are orthologs of PpeRGP. The molecular weights of AamRGP and AjaRGP are 5,156 and 5,117, respectively. The coding sequence (CDS) of AamRGP consists of 330 bp with an ORF encoding a peptide of 109 aa, including a signal peptide (26 aa), B-chain (20 aa), C-peptide (38 aa), and A-chain (25 aa) (Figure 1D) (DDBJ: LC040882) (38). The AjaRGP CDS is composed of 342 bp with an ORF encoding a peptide of 113 aa, comprising a signal peptide (26 aa), B-chain (20 aa), C-peptide (42 aa), and A-chain (25 aa) (Figure 1D) (DDBJ: LC104980) (39). The identity levels of the CDS in AamRGP and AjaRGP with respect to PpeRGP were 68 and 67%, although the homology between *AamRGP* and *AjaRGP* is 85%. The amino acid sequences of AamRGP and AjaRGP are not quite the same as that of PpeRGP. This suggests that the chemical structure of AamRGP is close to AjaRGP rather than PpeRGP (37).

**Figure 2:**
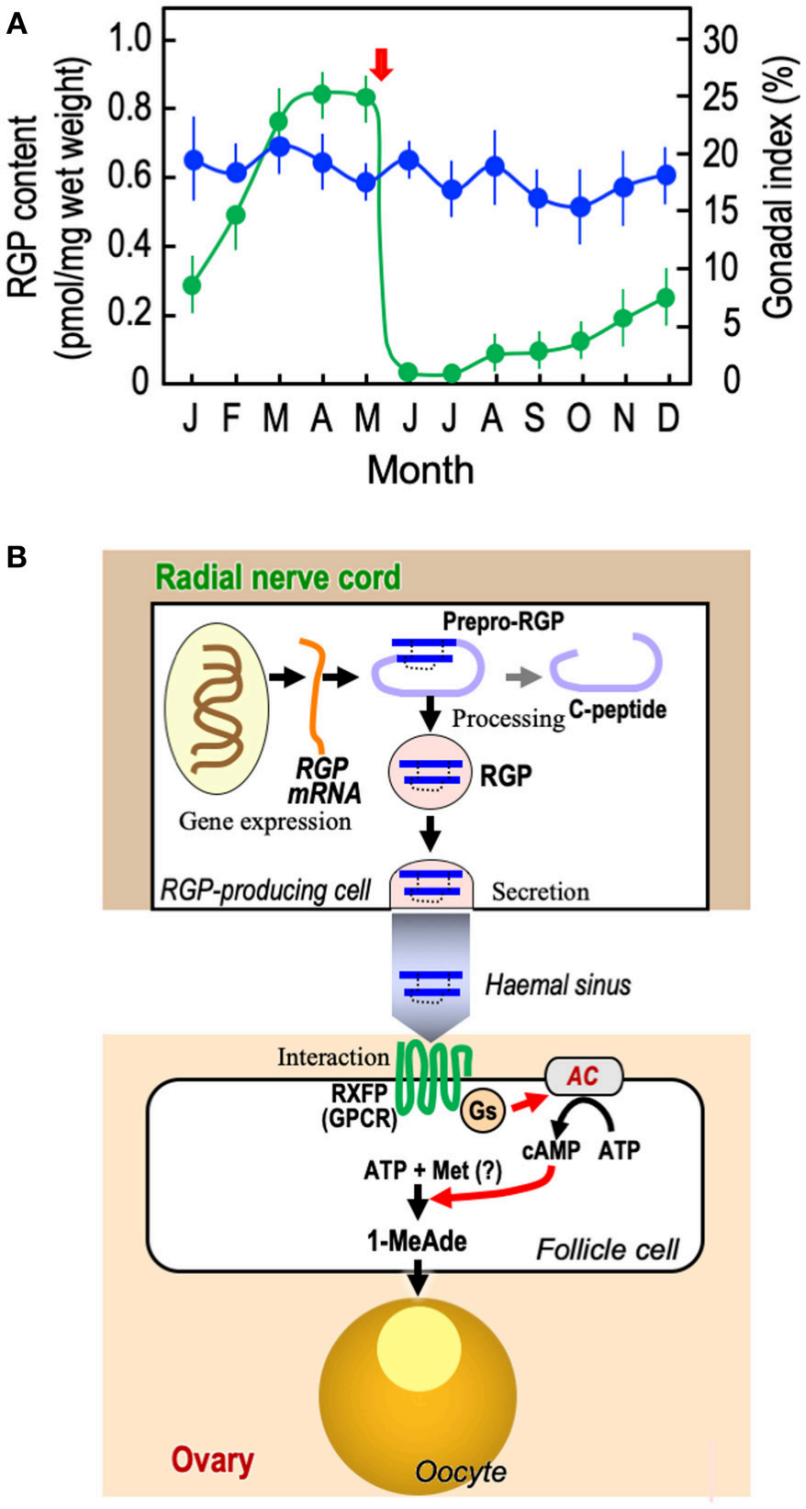
Overview of RGP. **(A)** Annual changes in RGP content in radial nerve cords and gonadal index in starfish *P. pectinifera*. Starfish *P. pectinifera* were collected monthly from Yokosuka (Kanagawa Prefecture, Japan). More than ten individuals (arm length 30–50 mm) per group were used for measurement of gonadal index (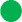) and the preparation of radial nerve cord extracts for the measurement of RGP content (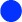). RGP contents were determined by ELISA (40). The gonadal index was calculated as the gonad weight per body weight × 100. Symbols and bars represent the mean of six independent samples and standard error of the mean (SEM), respectively. Arrow shows timing of spawning. **(B)** *De novo* synthesis, secretion, and hormonal action of RGP. AC, adenylyl cyclase; GPCR, G-protein-coupled receptor; Gs, G-protein as adenylyl cyclase stimulator; Met, methionine; and RXFP, relaxin-family peptide receptor.

As already introduced, the GSS seems to be present in the radial nerve cords throughout the year and its quantity is mostly the same irrespective of the breeding season (17, 18). This was confirmed by enzyme-linked immunosorbent assay (ELISA) of PpeRGP using anti-PpeRGP antibody (40). The result showed that PpeRGP contents remained almost constant regardless of the breeding or non-breeding season, although the breeding season of *P. pectinifera* estimated by the gonadal index (GI) values was around May in Yokosuka (Kanagawa Prefecture, Japan) (Figure 2A). It is assumed that the amount of RGP secreted upon spawning is considerably lower than that stored in radial nerve cords. On the basis of *in situ* hybridization, the mRNA of RGP is detected in the peripheral area of radial nerve cords proximal to the tube-feet, but not at the side of the haemal sinus (10, 41). Thus, RGP is *de novo* synthesized in the radial nerve cords and circumoral-nerve rings which are functionally equivalent to the central nervous system in vertebrates.

In the breeding season, RGP secreted from the radial nerve cords should be transported to the gonads (Figure 2B). Because it has been shown that RGP is capable of being released from the radial nerve cords in the presence of ionomycin (42), an increase in the level of intracellular Ca^2+^ is closely associated with RGP secretion. In contrast, a gonadotropin-releasing hormone-like peptide, pEIHYKVPGWGPG-NH_2_ (DDBJ: LC131035), of *P. pectinifera* fails to induce RGP secretion in isolated radial nerve cords (37). The mechanism of RGP secretion from radial nerve cords is still unknown.

RGP binds specifically to a membrane preparation of ovarian follicles in starfish (43, 44), and the follicle cells cultured with RGP show a dose-related increase in cAMP production, coinciding with an increase in 1-MeAde production (10, 44–46). The action of RGP is mediated through the activation of its receptor, leading to the activation of a G-protein and adenylyl cyclase in follicle cells (Figure 2B) (10, 46, 47). In this sense, RGP is functionally identical to vertebrate LH, especially piscine and amphibian LHs, acting on ovarian follicle cells to produce MIH to induce the final maturation or meiotic resumption of oocytes (48).

Previously, cross-species experiments using different species of starfish have shown that GSS generally acts non-species specifically, with some exceptions (15, 17, 18). There are three kinds of RGP orthologs, PpeRGP, AamRGP, and AjaRGP, among the class Asteroidea (Figure 1D) (37). Neither AamRGP nor AjaRGP induces spawning in ovarian fragments of *P. pectinifera* (38, 39), although PpeRGP can induce spawning in *A. amurensis* and *A. japonica* ovaries (39). Presumably, partial species-specificity observed in AamRGP and AjaRGP is caused by interaction with the receptor. Thus, chimeric RGP derivatives with replaced A- and B-chain from these RGPs were synthesized and examined whether to induce spawning. The results suggest that the B-chain of RGP plays an important role in the interaction with the receptor (49).

Receptors for the relaxin family peptides (RXFPs) belong to the superfamily of rhodopsin-like G-protein-coupled receptors (GPCRs) (50, 51). In humans, the relaxin superfamily consists of relaxin 1 (H1 relaxin), relaxin 2 (H2 relaxin), relaxin 3 (H3 relaxin), insulin-like peptide 3 (INSL3 also known as relaxin-like factor or Leydig insulin-like peptide), insulin-like peptide 4 (INSL4 or placetin), insulin-like peptide 5 (INSL5), and insulin-like peptide 6 (INSL6) (51–58). Previous studies have shown that H2 relaxin, INSL3, H3 relaxin, and INSL5 signal through RXFP1 (59), RXFP2 (60), RXFP3 (61), and RXFP4 (62) receptors, respectively. It is also considered that H1 relaxin is a ligand of the RXFP1 (63–65). However, the native receptors for INSL4 and INSL6 are yet to be identified (66). All the family members of relaxin peptides and their targets receptors have shown broad physiological roles (63–65). The activation of adenylyl cyclase by RXFP1 is complex and involves the interaction with several G-proteins, resulting in a biphasic pattern of cAMP accumulation (63). RXFP2 activates adenylyl cyclase *in vitro* but some physiological responses are sensitive to pertussis toxin (63). The signaling pathways activated by RXFP3 or RXFP4 result in the inhibition of adenylyl cyclase and a decrease in cAMP accumulation. Thus, it is possible that RGP receptor belongs to RXFP1/RXFP2 rather than RXFP3/ RXFP4, although the receptor has not been identified yet.

It has been demonstrated in mammals that the B-chain of relaxins and INSLs binds to the receptor (63–65). Despite its similarity with relaxin super family, however, the RGP sequence does not possess the vertebrate relaxin-specific receptor-binding cassette RxxxRxxI/V in the B-chain, a distinct and well-conserved feature of the relaxin group identified so far (67). A relaxin-like signaling system also exists in protostomes. Dilp8, *Drosophila* insulin-like peptide 8, exerts role in developmental transitions via a signaling pathway involving the LGR3, a leucine-rich repeat-containing G-protein coupled receptor, homologous to RXFP1 and RXFP2 (68). Because echinoderms belong to deuterostomes, starfish RGP and vertebrate relaxin are considered to be derived from the same ancestral peptide. Further studies on the receptor protein for RGP could provide useful insights into the hormonal action and evolution of species differentiation in the class Asteroidea.

## Data Availability

All datasets generated for this study are included in the manuscript and/or the supplementary files.

## Author Contributions

MM conceived the study and wrote the paper.

### Conflict of Interest Statement

The author declares that the research was conducted in the absence of any commercial or financial relationships that could be construed as a potential conflict of interest.
